# Winter chemical partitioning of metals bound to atmospheric fine particles in Dongguan, China, and its health risk assessment

**DOI:** 10.1007/s11356-019-05001-8

**Published:** 2019-04-09

**Authors:** Lin Huang, Yun-He Bai, Rui-Yue Ma, Ze-Ming Zhuo, Ling Chen

**Affiliations:** 1 School of Environment and Civil Engineering, Dongguan University of Technology, Dongguan, 523808 China; 2 Air Pollution Complex of Key Laboratory of Dongguan, Dongguan, 523000 China; 3 The Second Base of Science Research Institute of Metrology in Dongguan City, Dongguan, 523000 China

**Keywords:** Atmospheric particulates, Chemical forms, Exposure reaction, Health risks, Metal elements, Meteorological factors, PM_2.5_

## Abstract

To analyze the relationship between nanoparticles and the chemical forms in an urban atmospheric environment, metallic particles with different diameters were collected using a nanoparticle sampling system and analyzed for chemical and morphological characteristics, bioactivity, and the risk of carcinogenic and non-carcinogenic effects. The source of the atmospheric particles was analyzed based on the enrichment factor method, and the carcinogenicity of the atmospheric particles was analyzed using the health risk model. The partition contents of metals extractable by a weak acid, including As, Ca, Cd, Cs, Pb, Sr, and Zn, were in a range of 32.17–71.4%, with an average value of 47.07%. The content of oxides and reducible metals of all of the elements was generally low. Potassium was distributed mainly in the residual and weak-acid-extractable fractions. Barium had a high proportion of the oxidation state. Each fraction of Zn was basically the same, while the content of the weak-acid-extractable fraction was slightly higher. We found bio-access potential to be positively correlated with a high proportion of the weak acid extracts such as Mg, Sr, and Zn. We also found there to be a large weak-acid-extractable fraction (F1) and residual fraction (F4) and relatively enriched elements and strongly enriched elements, which means F1 and F4 may be the cause of enrichment. The hazard index (HI) and the total cancer risk (TCR) were far beyond the safety threshold when the diameter of the particle was in the range of 0.1–0.5 μm, indicating that the residents in Dongguan city were experiencing obvious non-carcinogenic and carcinogenic risks.

## Introduction

Atmospheric pollution is a serious global environmental problem. The main sources of atmospheric particulate matter, which may seriously damage the atmosphere, are automobile exhaust, industrial emissions, coal combustion, and road dust. Particles with different diameters have different compositions. Their surface contains adsorbed toxic and carcinogenic heavy metals and organic compounds, which represent a great hazard to human health. When the size of the particles is smaller than 2.5 μm, inhalation of these fine particles into the human body can lead to lung disease, heart disease, and arteriosclerosis (Ballester 2001; Donaldson et al. 2002; Lewis et al. 2005; Ostro et al. 2006; Samoli et al. 2005). Many epidemiological studies have shown that the lung cancer mortality rate increased by 8% and the mortality rate caused by cardiovascular disease increased by 6% when the concentration of PM_2.5_ increased to 10 g/m^3^ (Pope III et al. 2002). Compared with atmospheric coarse particles, PM_2.5_ has a proportionally larger surface to absorb pollutants from the atmosphere. The human body can absorb fine particles by respiration and accumulate heavy metals (Hu et al. 2012; Li et al. 2013b). In addition, the metal particles absorbed by atmospheric particle material (PM) can travel far and be deposited in the soil, bodies of water, and on leaves of plants through wet and dry sedimentation, thereby posing an environmental risk (Li et al. 2013b). Therefore, it is important to execute a human health assessment to study the harmfulness of air particulates with particles of different diameters.

The chemical composition of PM is crucial to its toxicity, as well as the particle concentration and size distribution (Xie et al. 2012). When assessing the potential environmental and health risks, the key is to know not only the amount of particle-bound metals but also their chemical partitioning, which determines the behavior of environmental metals (Betha et al. 2014). It is largely accepted that the chemical forms of metals determine their potential risks to human and environmental health through the processes of mobility and bioavailability (Pérez et al. 2008; Schleicher et al. 2011). Extraction methods that are run sequentially have been widely used to describe the chemical fractions of metals in environmental samples, including soil, river, and atmospheric particles under natural conditions (Arain et al. 2008; Davidson et al. 2006; Li et al. 2013a; Schleicher et al. 2011; Yuan et al. 2011). The human body is able to absorb and mobilize the weak-acid-extractable metal fractions (F1) in body fluids, producing toxicity (Mukhtar and Limbeck 2013). Components of metals that are bound to organic matter and oxidizable or sulfidic metals (F3) can become mobile under strong oxidization and can be converted or changed to the weak-acid-extractable (F1) or oxide fraction (F2). Toxicity of the residual fraction (F4) to animals and plants and its bioavailability are very low. Many studies have examined the relationship between meteorological factors and the chemical composition of PM, and there are some differences in the correlation between the particle concentrations and meteorology. In this study, we briefly discuss the correlation between the different metals and atmospheric meteorological factors.

Dongguan, China, has severe air pollution because of its rapid development. Therefore, local residents may face health risks, and it is necessary to establish measures for the management and control of airborne metals. In this study, we used certain assumptions for the health risk assessment that may introduce uncertainty in the evaluation model, reference data, toxic exposure default parameters, and population characteristics. Despite some uncertainty, the model has proven to be a useful tool for assessing risks to human health caused by toxic metal exposure in a city environment (Li et al. 2017).

There are few studies on the particle size distribution and health risk assessment in the existing research on heavy metals in Dongguan’s atmospheric particulate matter. The biological toxicity of some heavy metals in different particle sizes has also been rarely reported. Thus, the main objectives of this study were as follows: (1) to analyze the concentration of elements (Al, As, Ba, Ca, Cd, Cr, Co, Cu, Fe, K, Mg, Mn, Pb, Sr, V, and Zn) and the enrichment factors of metals in the atmospheric fine particles during the winter season of 2016–2017, (2) to analyze the chemical partitioning of metals bound to fine particles and determine the bio-accessibility of metals in PM_2.5_, and (3) to evaluate the carcinogenic and non-carcinogenic health risks via inhalation of toxic metals found in the atmospheric fine particles.

## Methods and materials

### Sampling

**Fig. 1:**
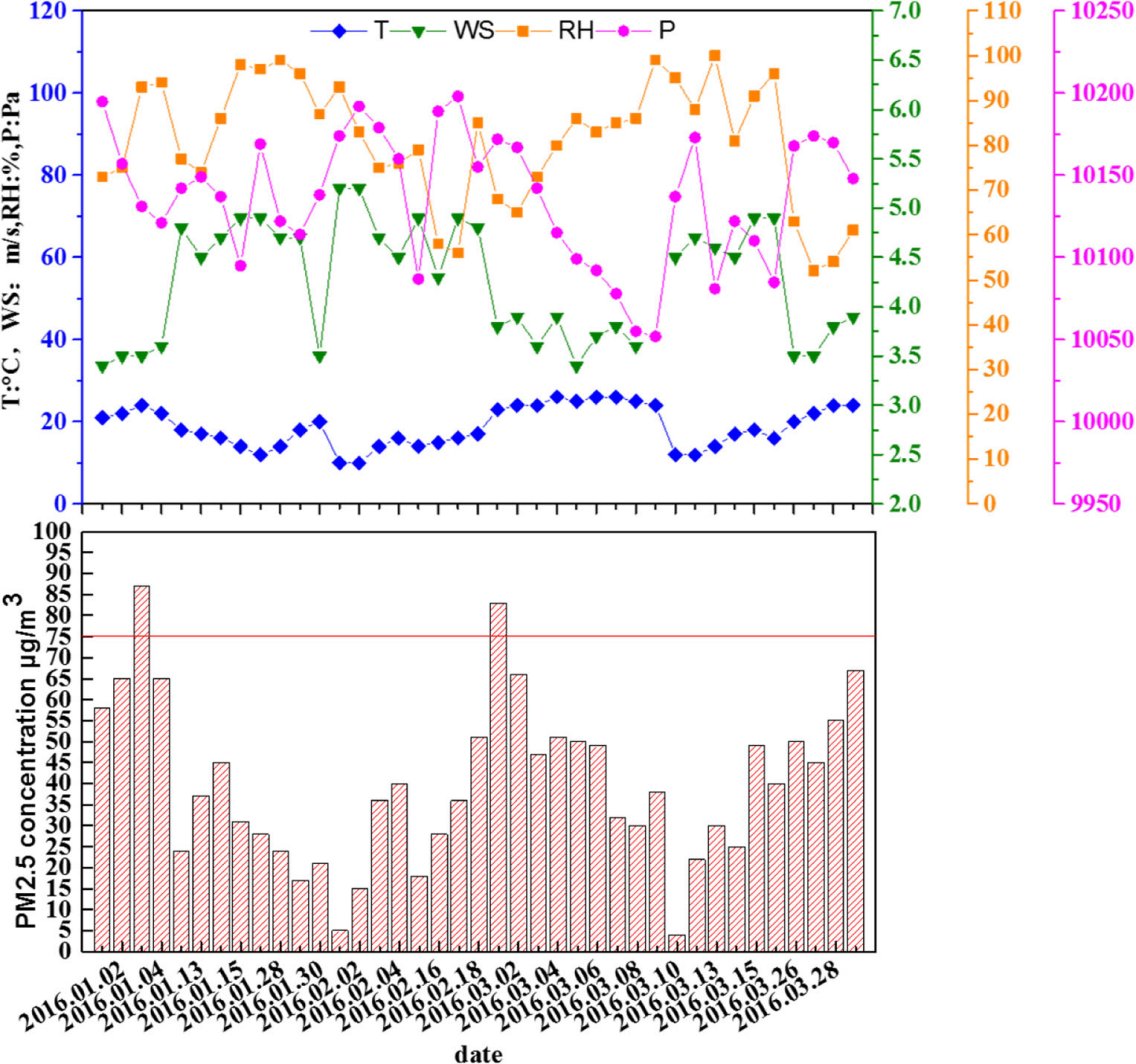
Meteorological data (P, pressure; RH, relative humidity; T, temperature; WS, wind speed) and concentration of PM_2.5_ measured during sampling

The city of Dongguan is located at 22° 39′–23° 09′ N 13° 31′–114° 15′ E, and is situated in the south of the province of Guangdong. The city is adjacent to the Pearl River Delta, and it is an important industrialized area in China. The atmospheric quality of the study region is influenced by the industrial emission of pollutants, human activities, and suburban traffic. The PM_2.5_ samples were collected from the Second Base of Science Research Institute of Metrology in Dongguan. Further details are illustrated in Fig. 1.

The PM_2.5_ samples were obtained using a six-level sampling apparatus, obtained from Westech Environmental, Inc. (Model M372106, flow rate 28.3 L/min, UK), that was used to collect particles with different diameters. The particle diameters were divided into six classes: I–VI (< 0.1 μm, 0.1–0.5 μm, 0.5–1 μm, 1–2.5 μm, and 2.5–10 μm, > 10 μm, respectively). Daytime and nighttime (8:00–18:00 and 19:00–7:00, respectively) sampling were implemented during the winter season from January 1 to 30, February 1 to 20, and March 1 to 29, 2016. There were a few days of fog and haze in this period. The air quality monitoring station near the sampling point also recorded the weather data per hour and the concentration of the air pollutants per hour. Before sampling, the filter membrane was conditioned in a desiccator for 48 h at 25 °C with a 60% relative humidity, and it was weighed. After sampling the aerosols, the filter membranes were conditioned using a desiccator for 48 h, and then they were weighed to determine the PM_2.5_ mass. A total of 42 samples were collected from January to March.

### Sequential extraction procedure

Four fractions of particulate-bound metals were obtained by the following four-step BCR (Community Bureau of Reference) procedure of sequential extraction: (1) weak-acid-extractable metals (F1); (2) reducible metals and oxides (F2); (3) organic matter-bound metals, sulfidic, and oxidizable metals (F3); and (4) residual metals (F4). Table 1 summarizes each of the steps. The modified BCR method was used to analyze the fractions of elements; 3/4 of the sample filter membrane, by area, was used in the test. In each extraction step, the samples were centrifuged (Shanghai Anting, TDL 500DR, China) for 40 min at 7500 rpm, and the supernatants were transferred to volumetric flasks. We used the filter to perform another extraction; we then centrifuged the extracted sample, and the supernatant was poured into the washing filter flask. The mixed supernatant was heated and concentrated to 1–2 mL and then diluted with 2% nitric acid (Shenzhen Chemical Co., Ltd., China) for analysis. The simultaneous analysis of six blank filters was conducted during the sequential BCR extraction procedure, and the metal concentration of each sample was adjusted by the subtraction of the blank average concentration.

**Table 1:** Operating conditions and reagents used in the BCR

Fraction	Reagents	Experimental setup
F1	0.11 mol/L CH3COOH	16-h agitation on a shaker at room temperature, centrifugal separation for 40 min at 7500 rpm×*g*; the supernatant was separated from the residuals
F2	0.1 mol/L NH_2_OH·HCl at pH 2	6-h agitation on a shaker and the washing process of insoluble matter was the same as before
F3	30% H_2_O_2_, 1 mol/L HAC at pH 3	Shaken for 60 min at room temperature, 1 h of heating in a water bath at low temperature (lower than 20 °C), heated until 2 mL of solution remained. The first steps were repeated. The sample was agitated on a shaker for 30 min. The supernatant was decanted from the residuals
F4	HNO_3_, H_2_O_2_	Shaken for 30 min at room temperature, dispelled in a microwave instrument. The supernatant was decanted from the residuals with a 0.45-μm needle filter.

### Analyses of the concentrations of the elements

The concentrations of the metals (Al, As, Ba, Cu, Ca, Cd, Cr, Co, K, Fe, Mg, Mn, Pb, Ni, Sr, V, and Zn) in the components and their chemical partitioning were measured using an inductively coupled plasma atomic emission spectrometer (ICP-AES, ICAP 7000, Thermo Fisher Scientific, USA) and inductively coupled plasma-mass spectrometer (ICP-MS, Agilent 7900, USA). The descriptive statistical correlation analysis of the experimental data was conducted using SPSS 21 software (IBM, USA). A mixed solution of 2% HNO_3_ and ^115^In at a concentration of 20 mg/L was utilized as the internal standard to measure the drift during the instrument calibration. Before the test, the ICP-MS needed to be calibrated for the analog signal/pulse ratios, dynamic linear range, and P/A factor. The calibration can expand the working curve and reduce the measurement time for the quantitative analysis. The internal recovery of each metal is the ratio between the sum of the concentrations of the 4 chemical fractions and the total metal concentration. The calculation of the recovery was as follows:

1 $$ \mathrm{Recovery}\ \left(\%\right)=\frac{F1+F2+F3+F4}{TC}\times 100\% $$

where F1–F4 represent the concentrations of the elements extracted from each fraction, whereas TC refers to their summed concentration. In general, recovery rates of the studied elements ranged from 86 to 112%, showing that the total content of these 4 fractions and the total concentration are in good agreement, reliable, and repeatable. In this study, the heavy metal recovery rate ranged from 87 to 101%, indicating that our experimental results are consistent with the references.

### Enrichment factors

The enrichment factor (EF)—a test of elements based on the specification of the conservative reference value (Chen et al. 2008)—was utilized to identify the possible origins of the elements in atmospheric fine particles. The enrichment factor method was utilized to indicate the degree of enrichment of the elements in the atmospheric PM and evaluate the particulate matter in natural and man-made sources. The enrichment factor was calculated as follows:

2 $$ E{F}_i=\frac{{\left({C}_i/{C}_n\right)}_{\mathrm{sample}}}{{\left({C}_i/{C}_n\right)}_{\mathrm{reference}}} $$

*C*_i_concentrations of i elements,

*C*_n_reference element concentration.

The enrichment factor method is a calculation of double normalization. It can eliminate the influence of the uncertain factors caused by atmospheric particulate sampling, analysis, wind speed, wind direction, and the distance from a variety of sources of pollution. It is thus more reliable and accurate than a direct comparison of the element concentrations (Shao et al. 2006). Generally, the reference elements are selected from the Earth’s crust in a region with few pollution sources and have a good chemical stability; thus, they provide a high accuracy of the analysis results of poorly volatile elements. Many studies used reference elements such as Fe, Al, Si, Ti, and Se. For the purposes of the present study, we chose the reference element Al, and we used the values from the published Chinese values of background soil elements (CNEMC 1990).

The greater the EF value, the higher the enrichment. We divided the results of the EF analysis into 3 categories: the elements with EF > 10 were mainly derived from anthropogenic pollution; the elements with EF < 10 originated from a natural source, mainly from soil background materials, and a low EF indicated that the target element was scarce. The elements with EF = 10 were a result of interactions between anthropogenic and natural sources (Yang et al. 2012). In addition, 10 < EF < 100 indicated that the element was relatively abundant, while the EF > 100 indicated a typical abundance of the element in question.

### Assessment of the health risks of toxic metals found in the atmospheric fine particles

The health risk assessment model of human exposure recommended by the US EPA is an important method in the field of heavy metal health risk research. We used the concepts of the average daily dose (ADD), carcinogenic risk (CR), and non-carcinogenic risk value (hazard quotient, HQ) proposed by the model and selected the exposure parameters of the specific population. Air pollutants enter the human body mainly through respiratory pathways. However, if we only consider the health risks caused by respiratory exposure and ignore the health risks caused by hand-mouth intake and skin contact, the calculated health risks will be less than the actual health risks. Therefore, 3 types of exposure were included in this study. The most commonly used exposure equation is the average daily dose (ADD). There are 3 main ways that atmospheric particles enter the bodies of humans: dermal contact (D_der_, mg kg^−1^ day^−1^), ingestion (D_ing_, mg kg^−1^ day^−1^), and inhalation (D_inh_, mg kg^−1^ day^−1^). The calculations for these values were as follows:

3 $$ {\mathrm{ADD}}_{\mathrm{ing}}=\frac{C\times \mathrm{IngR}\times \mathrm{EF}\times \mathrm{ED}\times \mathrm{CF}}{\mathrm{BW}\times \mathrm{AT}} $$

4 $$ {\mathrm{ADD}}_{\mathrm{dermal}}=\frac{C\times \mathrm{SA}\times \mathrm{AF}\times \mathrm{ABS}\times \mathrm{EF}\times \mathrm{ED}\times \mathrm{CF}}{\mathrm{BW}\times \mathrm{AT}} $$

5 $$ {\mathrm{ADD}}_{\mathrm{inh}}=\frac{C\times \mathrm{InhR}\times \mathrm{EF}\times \mathrm{ED}}{\mathrm{PEF}\times \mathrm{BW}\times \mathrm{AT}} $$

The risk characterization can be categorized into the carcinogenic risk assessment and assessment of the non-carcinogenic risk. The HQ represents the non-carcinogenic risk. The expression of the assessment of the carcinogenic risk was created using the following formulas:

6 $$ \mathrm{HQ}=\frac{\mathrm{ADD}}{\mathrm{RFD}} $$

7 $$ \mathrm{HI}={\sum}_{i=1}^n{\mathrm{HQ}}_i $$

8 $$ \mathrm{CR}={\mathrm{ADD}}_{\mathrm{inhalation}}\times {\mathrm{CSF}}_{\mathrm{inhalation}} $$

where *C* represents the concentration of the particulate matter, IngR is the intake rate of ingestion (30 mg/day for adults and 60 mg/day for children), EF represents the frequency of exposure (180 days/year), and ED is the exposure duration (for children of 6 years and adults of 24 years). CF is the conversion coefficient (10^−6^ kg/mg). ATN represents the average time (non-carcinogens: ATN = ED × 365 days × 24 h per day; carcinogens: ATN = 70 years × 365 days per year × 24 h). SA represents the parameter for the skin surface (the adult threshold is 5700 cm^2^ and the child threshold is 2700 cm^2^). AF is the adsorption coefficient of skin to soil (0.07 mg/cm^2^ for adults and 0.2 mg/cm^2^ for children). ABS is the skin absorptivity (0.001). InhR is the respiratory rate of inhalation (7.63 m^3^/day for adults and 20 m^3^/day for children). PEF is the emission factor (1.36 × 10^9^ m^3^/kg), RFD represents the reference dose (mg/kg/day), and the cancer slope factor (mg/kg/day) is represented by CSF.

We measured the total concentration of Cr. However, the US EPA lists Cr (VI) within group A (human carcinogens) and Cr (III) in group D (not classified as a human carcinogen). An assumption was made that the ratio of the concentration of Cr (VI) and Cr (III) ranges from 1 to 6, according to the US EPA (2011), and other researchers used this ratio to assess the health risk of Cr (VI) in PM_2.5_ (Hsu et al. 2016). For the purpose of the EC estimation in the present study, the Cr (VI) concentration was therefore assumed to be 1/7th of the Cr total concentration.

## Results and discussion

### PM_2.5_ mass concentration

The quality of air in Dongguan area was analyzed using the air quality index (AQI) and the National Ambient Air Quality Standards (NAAQS; GB3095-2012). The average concentration of PM_2.5_ was 40.1 m/m^3^, with a range of 4–87 μg/m^3^, while the highest PM_2.5_ concentration, detected on January 3, was 87 μg/m^3^, which was 1.16 times the limit of the air quality standards. The relative humidity was 95% (the highest value measured), the temperature was 24 °C, and the sampling speed of the wind was only 3.5 m/s.

On haze-fog (HF) days, the average wind speed was 3.65 m/s (range 3.5–3.8 m/s) and the relative humidity was 80.5% (range 68%–93%; Fig. 1). During the period of HF (January 3 to March 1), the average daily concentration of PM_2.5_ was 85 μg/m^3^ (range 83–87 μg/m^3^), which exceeded the 24-h limit (75 μg/m^3^) specified by NAAQS and GB3095-2012 (Fig. 1). The high concentration of PM_2.5_ may be an indicator for HF quantification. On non-HF days, the average wind speed was 4.26 m/s (range 3.4–5.2 m/s) and the relative humidity was 81.03% (range 52–100%).

### Total concentration of elements

As shown in Table 2, Zn, Fe, Al, Ca, and K had high concentrations, while the concentrations of Cd, V, Sr, and Co were lower. In general, the average concentrations of all of the elements were significantly elevated in winter, especially on the HF days. This result is mainly because the atmosphere during HF days is layered, stable, and conducive to air pollutant accumulation (Kang et al. 2013). We referred to the concentration limits set by NAAQS and WHO as standards in the metal concentration analysis, and we noted that both of them were applicable to the limit of the metal particle concentration (Li et al. 2013a). During the sampling period, the As total concentration exceeded the limit values of NAAQS (6 ng/m^3^) and WHO (6.6 ng/m^3^); the same was true for Cd (the limit value of both WHO and NAAQS is 5 ng/m^3^). However, the concentration of the sampled Pb reached one-half of the limit values of WHO and NAAQS for Pb (500 ng/m^3^). NAAQS defines no limit values for Ni, Mn, and V; however, the concentration of Ni exceeded the WHO guideline (25 ng/m^3^) by tenfold, and Mn exceeded twofold the WHO guideline (150 ng/m^3^) during the sampling period, which illustrates that the two metals may pose a certain risk to human health. The concentration of V was below the WHO limit values (1000 ng/m^3^), illustrating that V was relatively scarce in the atmosphere and did not represent a health risk.

**Table 2:** Metal enrichment factors and total concentrations in fine particulate matter with different diameters during the sampling period

Metal	TC (ng/m^3^)	EF				The EF of PM_2.5_
		d < 0.1	0.1 < d < 0.5	0.5 < d < 1	1 < d < 2.5	Mean
Al	2350.00 ± 80.00	/	/	/	/	/
As	75.80 ± 2.53	208.8 ± 68.96	141.6 ± 44.06	266.1 ± 281.85	112.19 ± 82.13	786.87 ± 387.77
Ba	504.00 ± 7.966	18.6 ± 8.03	7.1 ± 4.58	12.1 ± 5.56	12.44 ± 9.34	52.61 ± 14.48
Ca	3238.8 ± 104.7	5.1 ± 3.19	1.4 ± 1.31	3.4 ± 1.67	3.5 ± 3.28	13.17 ± 9.03
Cd	7 ± 0.5	434.5 ± 236.26	486.13 ± 173.02	451.5 ± 281.64	444.5 ± 605.31	2180.22 ± 1127.70
Co	35.65 ± 1.48	22.3 ± 17.53	503.7 ± 502.02	11.9 ± 5.29	15.03 ± 10.80	864.45 ± 657.37
Cr	1748.3 ± 126.3	52.7 ± 36.05	2403.5 ± 2488.84	24.7 ± 11.85	36.1 ± 33.37	3973.62 ± 3330.15
Cu	308.4 ± 12.9	142.69 ± 71.00	261.88 ± 165.94	119.09 ± 83.61	88.79 ± 67.41	612.4543908 ± 65.64
Fe	2797.9 ± 75	3.16 ± 1.55	8.7 ± 14.11	2.6 ± 0.97	3.3 ± 1.77	768.09 ± 203.60
K	5970. ± 216.	6.5 ± 3.61	3.0 ± 0.79	4.5 ± 1.51	2.95 ± 2.46	17.14 ± 7.36
Mg	1700.3 ± 81.7	1.1 ± 0.56	0.5 ± 0.44	0.978 ± 0.38	0.99 ± 0.50	3.46 ± 1.23
Mn	301.3 ± 24.3	3.6 ± 1.46	21.5 ± 22.48	3.07 ± 0.77	3.01 ± 1.93	43.23 ± 22.05
Ni	1112.8 ± 64.8	86.4 ± 30.98	1669.4 ± 663.03	52.8 ± 20.92	64.4 ± 56.15	2846.74 ± 703.85
Pb	204.4 ± 8.7	122.8 ± 60.00	79.8 ± 22.97	120.4 ± 68.91	80.48 ± 81.19	445.11 ± 156.22
Sr	57.7 ± 2.1	7.96 ± 4.41	3.15 ± 2.58	7.56 ± 2.53	6.23 ± 4.51	23.10 ± 9.37
V	27.95 ± 2.3	1.84 ± 0.97	9.7 ± 10.19	2.2 ± 1.20	2.8 ± 2.56	22.50 ± 12.02
Zn	3591.6 ± 51.84	796.3 ± 410.26	283.9 ± 161.44	652.5 ± 296.49	560.96 ± 465.14	2284.92 ± 932.38

Some of our conclusions are confirmed by the information shown in Table 3, indicating that there is a certain relationship between the concentrations of the metal elements and the meteorological factors. In this study, the concentrations of most elements (except Mg and Zn) were negatively correlated with the temperature (the correlation coefficients range from − 0.080 for Ca to − 0.729 for K); the reason for this correlation was that the airborne particles were in the low frequency range of the temperature inversion when the temperature was high. Even if the temperature inversion happened, the thickness of its inversion layer was low, the strength was weak, the boundary layer structure was unstable, and, under the effect of the diffusion and settlement, the concentration of the atmospheric particles decreased. However, the concentrations of other metals (except Cu and Ca) were negatively correlated with the relative humidity, especially the concentration of K (*P* < 0.01). As the relative humidity increases, it not only inhibits the rise of the coarse particles but also helps the atmospheric particles to form a coagulant, promoting precipitation and reducing the concentration in the air. Most of the total metal concentration was positively related to the speed of wind. Generally, the greater the wind speed, the more the wind enhances the diffusion and dilution of the air pollutants. An extended lack of air flow will inhibit pollutant diffusion and increase the pollution in the near-ground layer. A high wind speed can promote the exchange and migration of the atmosphere, but when the wind speed is too great and the climate is dry, it will lift sand into the air, aggravating the pollution. Our study confirms this outcome.

**Table 3:** Relationship between the total concentrations of the metal and meteorological factors as represented by Pearson’s coefficient (r)

	T	WS	RH	P	PM20.5	SO2	NOx	CO
Al	− 0.571**	− 0.479	− 0.334	0.216	0.150**	0.119**	0.478**	0.634**
As	− 0.291	0.143	− 0.331	0.830**	0.012	0.037	0.139	0.252
Ba	− 0.207	− 0.124	− 0.617	0.182	0.067	0.468**	0.124	0.768**
Ca	− 0.080	0.067	0.373	0.751**	0.541**	0.478**	0.185	0.754**
Cd	− 0.322**	0.134	− 0.135	0.829**	0.214	0.389**	0.270	0.810**
Co	− 0.416**	0.269	− 0.397	0.879**	0.280	0.159	0.412**	0.573**
Cr	− 0.367**	0.169	− 0.138	0.806**	0.259**	0.440**	0.293	0.872**
Cu	− 0.590**	0.511	0.413	0.193	0.178	0.101	0.548**	0.687**
Fe	− 0.214	0.050	− 0.570	0.979**	0.350**	0.208**	0.176	0.433**
K	− 0.729**	− 0.709	− 0.840**	0.077	0.619**	0.163	0.668**	0.548**
Mg	0.492**	− 0.453	− 0.781	0.086	0.357**	0.122	0.502**	0.733**
Mn	− 0.142	0.036	− 0.420	0.761**	0.484**	0.281	0.317	0.614**
Ni	− 0.504**	0.307	− 0.179	0.867**	0.131**	0.273**	0.364**	0.734**
Pb	− 0.161	0.011	− 0.374	0.865**	0.373**	0.320**	0.221	0.632**
Sr	− 0.652**	− 0.580	− 0.553	0.242	0.278	0.060	0.671**	0.795**
V	− 0.172	0.055	− 0.733	0.937**	0.398**	0.047	0.199	0.203
Zn	0.286	− 0.127	− 0.184	0.281	0.166	0.532**	0.144	0.829**

**Correlation is significant at *P* = 0.01 (2-tailed). * Correlation is significant at *P* = 0.05 level (two-tailed). *RH*, relative humidity; *P*, pressure; *T*, temperature; *WS*, wind speed

### Sources of metal and the enrichment factor

**Fig. 2:**
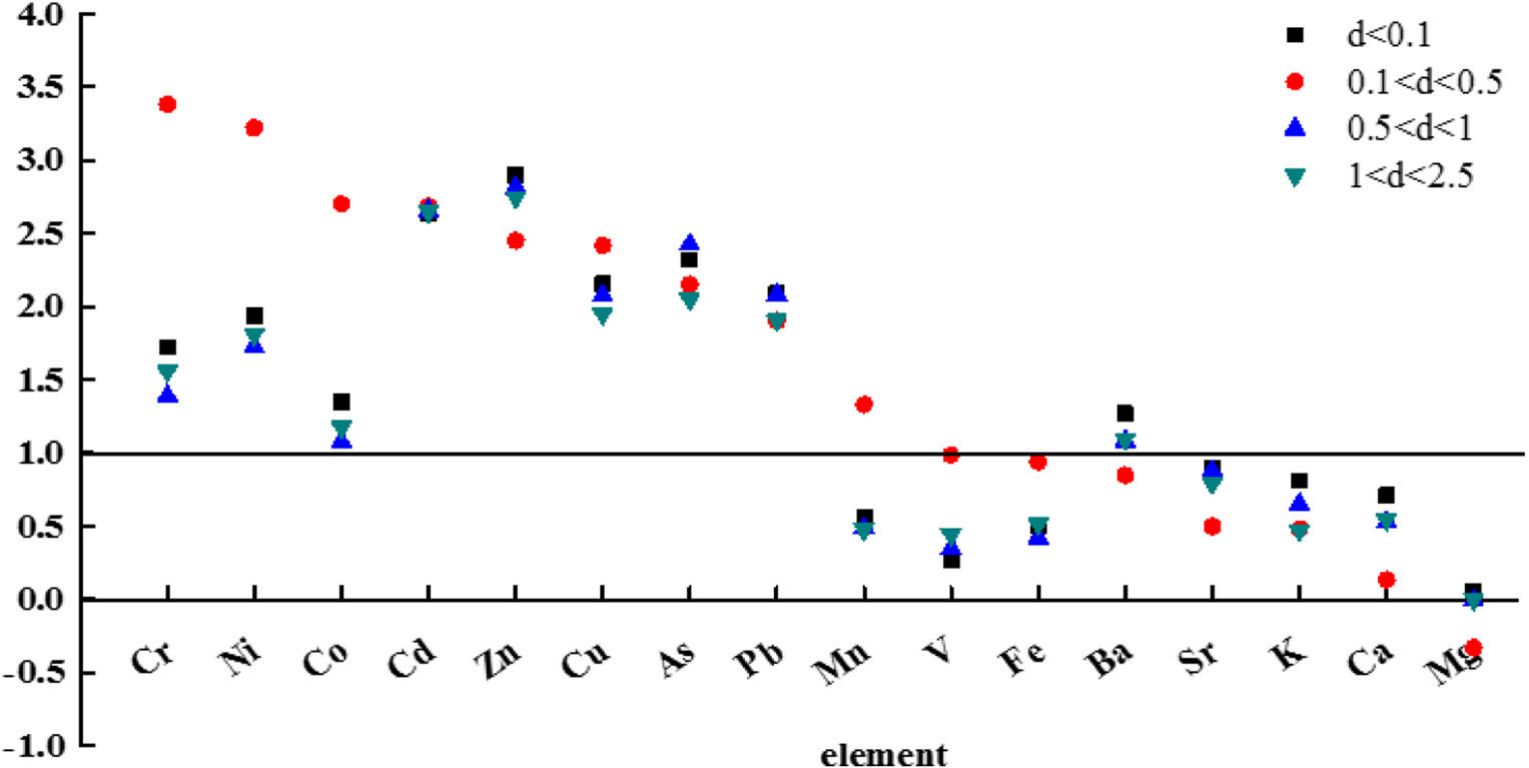
A logarithmic diagram of the enrichment factors

The average EF values for different elements on every sampling date are shown in Table 2. The EF average values for K, Ca, Fe, Mg, Sr, and V were lower than 10, indicating no enrichment of these metals, and that they originated mainly from sources in nature, such as in soil materials blown into the atmosphere and rocks undergoing natural weathering. The EF average values of Cr, Co, Cu, and Ni were between 10 and 100, indicating a moderate enrichment for these metals. The EF values for the remaining metal elements exceeded 100, indicating the significant enrichment of these metals. The metal with the highest EF value in this study was Cr (its average EF was 2517). In addition, a logarithmic diagram of the enrichment factors (Fig. 2) was made to visualize the relationship between the different particle diameters and the enrichment of the elements in an intuitive way. It can be seen that between the boundaries 1 and 2, the source of some of the particle sizes was the result of the combined action of an anthropogenic source and natural source. Particles with a diameter of 0.1–0.5 μm were strongly enriched with Cr, Co, and Ni. The enrichment factor of Zn and Cu was basically the same at different particle diameters, while Cd and Pb were mainly concentrated in the particle diameter range of d_p_ < 1 μm. Overall, the smaller the particle diameter was, the higher the possibility that the element originated from natural sources. When the element originated from both natural and anthropogenic sources, the particles (d < 0.1 μm) had a high EF. When the element was strongly enriched, the particles with a diameter of 0.1–0.5 μm predominated.

Scholars have made some achievements in the apportionment of atmospheric particulates in the sources of heavy metals. Coal fly ash, industrial emissions, exhaust emissions, metal smelting plants, the ceramic industry, the mining industry, waste incineration (Duan and Tan 2013), energy power stations, the cement industry, and transportation are sources of atmospheric particles with metal (Lahd Geagea et al. 2008). Table 3 shows that the concentrations of most elements correlate positively with NO_2_, CO, and SO_2_ emissions, which are mainly the result of coal combustion.

### Chemical partitioning of metals bound to fine particles

**Fig. 3:**
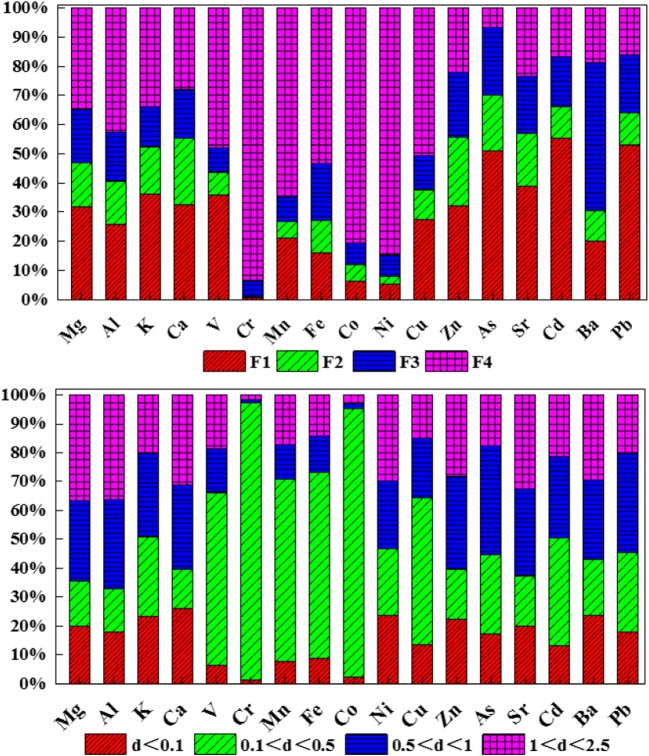
PM_2.5_ metal chemical partitioning. Metal chemical partitioning at different particle diameters and during the sampling period

The concentrations and chemical partitioning of the studied metals in the four fractions are presented in Fig. 3. Geochemical elements (EF < 10) like K, Ca, Sr, and V existed mainly in the F1 part. Mg and Fe were mainly found in the fraction F4, with the average shares of 41.6% and 34.9%, respectively. In addition, the soluble and exchangeable fractions (F1) of As, Cd, and Pb exceeded those of other metals, over 50%, while the average share of the same three elements in F4 was very low. As was present in a relatively high proportion of 93.38% in the first three fractions. Cr, Co, and Ni were present in the last three fractions in relatively high proportions of 99.32%, 93.75%, and 94.5%, respectively. Cr, Co, Mn, and Ni were predominantly present in fraction F4.

As shown in Fig. 3, Fe and Mg originated from natural sources (EF < 10) and their F1 to F4 ratio was high, indicating that the F1 and F4 fractions contributed significantly to the environmental behaviors of the metals. The EF (10–100) was in Cr, Ni, Co, Cu, and Pb, and the ratio of F1 in Pb was high. Ba was mostly present in the F3 fraction, while the ratio of F1 and F4 was high for K, Ca, and V. Fe and Mn were present predominantly in the F4 fraction, and Sr was found mainly in the F1 fraction. The values of EF of As, Cd, Co, Cr, Ni, Pb, and Zn exceeded 100, indicating a significant enrichment. As, Cd, and Pb predominated in the F1 fraction, while Ni, Co, and Cr predominated in the F4 fraction. The ratio of F1 to F4 was high for Zn. In conclusion, F1 and F4 were the key fractions, containing relatively enriched and highly enriched elements. Figure 3 shows that metals V, Cr, Mn, Fe, Co, and Ca were mainly found with particle sizes of 0.1–0.5 μm. The other elements were distributed uniformly in 4 particle sizes.

### Bio-accessibility of metals in PM_2.5_

The proportion of F1 can be used to study the bioactivity of various metals (Li et al. 2017). We divided the particle sizes into four levels to evaluate the potential bioactivity risk of the different metals.

**Fig. 4:**
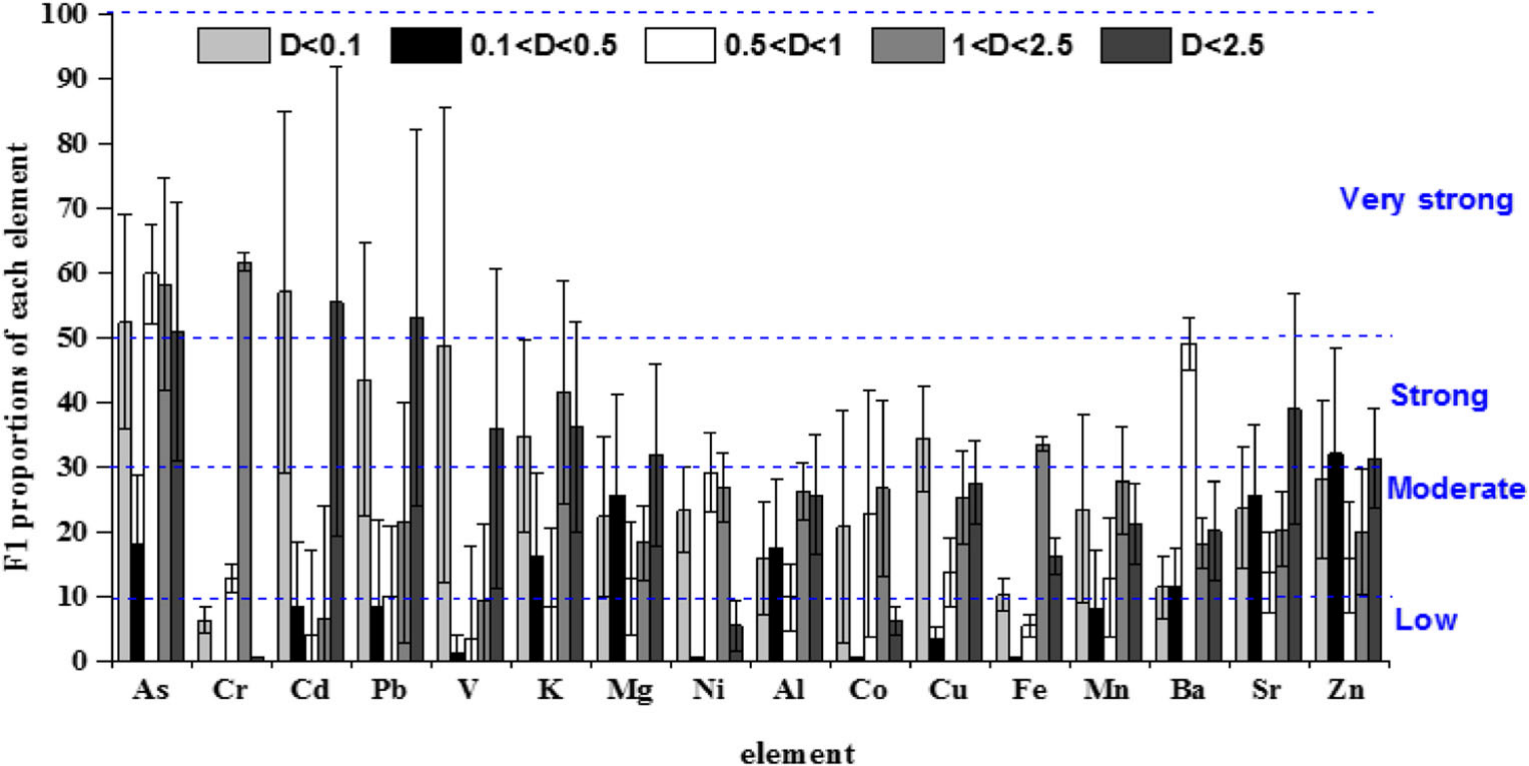
Percentage of F1 and bioactivity found during the sampling period

The percentages of F1 and bio-accessibility during the sampling period are shown in Fig. 4. We found that As, Cd, and Cr metals had the highest potential biological activity. The highest average proportion of Cd, 57%, was present in the F1 fraction when the particle diameter was d_p_ < 0.1 μm. The highest average proportions of As with particle diameters of 0.1–0.5 μm and 0.5–1 μm in the F1 fraction were 18.2% and 59.8%, respectively. The highest average proportion of Cr in F1 was 61.6% when the particle diameter was 1–2.5 μm, while the F1 of Cr with d_p_ < 2.5 μm was almost 0. We found that Cu, Pb, and V with d_p_ < 0.1 μm, Fe with d_p_ 1–2.5 μm, K in each size, except d_p_ 0.5–1 μm, and Zn with 0.1–0.5 μm had a relatively strong bio-accessible risk. Finally, Cr, Co, Cd, Cu, Fe, Pb, K, V, and Ni posed a low bio-accessible risk in particles with a diameter of 0.1–0.5 μm.

The F1 proportion of Mg, Sr, and Zn with different particle diameters was at a low potential risk level, but when the concentration of d_p_ < 2.5 μm exceeded the original basis, it rose to a strong risk level, indicating that the potential risk was not stronger when the particle size was small. Similarly, we found that the potential risk of some metals, such as Co, Fe, and Ba, dropped suddenly at d_p_ < 2.5 μm. Therefore, we speculated about the chemical properties of each metal and determined that the potential risks vary at different particle diameters.

There is some inconsistency in regard to suitable solvents used for determining the accessibility of metals to organisms. Pure water can act as a useful agent for leaching, and it is used for the calculation of PM in metal biodegradable points (Mukhtar and Limbeck 2013). Table 4 compares the results of this study with those of other studies. The F1 fractions of PM_2.5_ generally vary among different cities, which may be due to the composition of PM_2.5_, sampling period, and different sources of metals in the air. Among the cities studied, the highest average share of Cd in F1 was found in Dongguan city. Al and Fe were found in lower F1 proportions of other cities.

**Table 4:** Comparison of the percentage of F1 in PM_2.5_ using water as a leaching agent and the associated references

	Schleicher et al. (2011)	Falta et al. (2008)	von Schneidemesser et al. (2010)	Feng et al. (2009)	Li et al. (2017)	Heal et al. (2005)	This study (2017)
Sampling site	Beijing, China	Vienna, Austria	Lahore, Pakistan	Guangzhou, China	Nanjing, China	Edinburgh, UK	Dongguan, China
Extraction time (min)	180	120	?	180	180	60	960
T (°C)	RT		RT	RT	RT	RT	RT
Particle	PM_2.5_	PM_2.5_	PM_2.5_	PM_2.5_	PM_2.5_	PM_2.5_	PM_2.5_
Unit	%	%	%	%	%	%	%
Al	8		1.5 ± 1.0	1.5	9.1 ± 3.4		51.9
As	52		36 ± 23	32.6	35 ± 8.1	64	84.8
Ba			30 ± 9.6		36 ± 7.8		54.8
Ca	27		76 ± 15		50 ± 9.5		55.7
Cd	34	86	73 ± 17	39.7	46 ± 6.8	75	89.9
Co	40	44	22 ± 5.7	26.8	17 ± 7.5		54.22
Cr	28	18	9.6 ± 6.6	6.6	35 ± 7.7	28	65.98
Cu	17	78	13 ± 8.4	20.1	37 ± 10	50	59.61
Fe	7		1.8 ± 1.3	1.7	11 ± 5.0	10	57.84
K	54		67 ± 15		62 ± 13		47.57
Mg	38		22 ± 5.7		45 ± 7.5		58.20
Mn	44	56	37 ± 9.4	30.3	38 ± 7.9	45	38.50
Mo		44	37 ± 8.5	45.2	54 ± 13		
Na			79 ± 10		59 ± 7.7		
Ni	4	64	38 ± 19	27.6	29 ± 8.4	45	88.08
Pb	20	82	23 ± 16	12.2	28 ± 5.9	50	56.52
Sr	20		65 ± 13		56 ± 8.0		
Ti	2	2	0.3 ± 0.2		3.5 ± 2.5	14	
V	21		19 ± 15		29 ± 11	80	74.40
Zn	20	74	65 ± 22	46.3	54 ± 8.8	75	71.47

### Health risk assessment

The health risk of inhalation was analyzed for an assessment of the adverse effects of airborne metal exposure. As shown in Table 5, the values of non-carcinogenic risk for adults resulting from inhaling atmospheric particles containing Cd, Cr, Cu, Mn, Ni, Pb, and Zn were lower than the safety limit (HI < 1), while adults (4.69E+01) and children (3.64E+02) had the greatest risk of non-carcinogenesis by inhaling atmospheric particles (0.5–2.5 μm). In addition, the concentrations of the metals Cr, Mn, Ni, and Pb contributed significantly to the non-carcinogenic risks, illustrating that the concentrations of these toxic metals exceed safety levels. Overall, the non-carcinogenic hazard index (HI) of atmospheric particulate matter in Dongguan city exceeded 1, indicating that there, the residents were experiencing obvious non-carcinogenic risk; the government needs to take measures to control the quality of the air.

**Table 5:** Health risks of different particle sizes

						d < 0.1 μm			0.1–0.5 μm			0.5–1 μm			1–2.5 μm	
						Children	Adults		Children	Adults		Children	Adults		Children	Adults
Metal	RFD-ADD_ingestion_	RFD-ADD_dermal_	RFD-ADD_inhalation_	RFD-CSF_inhalation_	C (mg/kg)	HI	HI	C (mg/kg)	HI	HI	C (mg/kg)	HI	HI	C (mg/kg)	HI	HI
Non-carcinogenic																
Zn	3.00E-01	6.00E-02	3.01E-01		34,694.89	2.39E-01	2.61E-02	2.12E+04	1.46E-01	1.59E-02	26,853.28	1.85E-01	2.02E-02	30,214.26	2.08E-01	2.27E-02
Pb	3.50E-03	5.25E-04	3.52E-03		1861.36	1.11E+00	1.22E-01	1.69E+03	1.01E+00	1.11E-01	1638.13	9.81E-01	1.08E-01	1446.52	8.66E-01	9.51E-02
Cd	1.00E-03	1.00E-05	1.00E-03		51.92	1.98E-01	2.56E-02	8.06E+01	3.07E-01	3.97E-02	48.06	1.83E-01	2.37E-02	63.56	2.42E-01	3.13E-02
Ni	2.00E-02	2.06E-02	5.40E-03		3197.64	3.19E-01	3.43E-02	2.11E+05	2.11E+01	2.26E+00	747.36	7.44E-02	8.01E-03	1129.57	1.13E-01	1.21E-02
Mn	4.60E-02	1.43E-05	1.84E-03		1207.95	1.61E+00	2.43E-01	1.23E+04	1.64E+01	2.48E+00	801.55	1.07E+00	1.61E-01	1024.54	1.36E+00	2.06E-01
Cr	3.00E-03	2.86E-05	6.00E-05		4820.59	6.31E+00	8.17E-01	2.48E+05	3.24E+02	4.20E+01	854.94	1.12E+00	1.45E-01	1667.49	2.18E+00	2.82E-01
Cu	4.00E-02	4.02E-02	1.20E-02		1753.46	8.73E-02	9.39E-03	6.03E+03	3.01E-01	3.23E-02	1444.21	7.19E-02	7.74E-03	1393.47	6.94E-02	7.46E-03
Sum						9.88E+00	1.28E+00		3.64E+02	4.69E+01		3.68E+00	4.73E-01		5.05E+00	6.57E-01
Carcinogenic						CR	CR		CR	CR		CR	CR		CR	CR
Cd				6.30E+00	0.05	1.36E-11	4.43E-12	8.06E+01	2.10E-08	6.88E-09	48.06	1.25E-08	4.10E-09	63.56	1.66E-08	5.43E-09
Ni				8.40E-01	3.20	1.11E-10	3.64E-11	2.11E+05	7.36E-06	2.41E-06	747.36	2.60E-08	8.51E-09	1129.57	3.93E-08	1.29E-08
Cr				4.20E+01	4.82	8.39E-09	2.74E-09	2.48E+05	4.31E-04	1.41E-04	854.94	1.49E-06	4.87E-07	1667.49	2.90E-06	9.49E-07
TCR						8.52E-09	2.78E-09		4.39E-04	1.43E-04		1.53E-06	4.99E-07		2.96E-06	9.67E-07

In the assessment of carcinogenic risk, a value of the carcinogenic risk between 1E-06 and 1E-04 indicated an acceptable risk of carcinogenesis, 1E-04 indicated a high carcinogenic risk environment, and 1E-06 represented a precautionary criterion. The carcinogenic risks of inhalation of Cd, Cr, and Ni (d_p_ < 0.1 μm) from the air were lower than the children’s precautionary criterion (10^−6^), indicating that the amount of heavy metals contained in the atmospheric particles at this level was not enough to cause carcinogenic risk. The risk of carcinogenesis for children from inhaling atmospheric particulate matter (0.5–2.5 μm) just exceeded the warning line (10^−6^), while that of adults did not exceed the warning line, suggesting that children inhaling heavy metal particles in the two grade particles can cause a slight risk of carcinogenesis. However, when adults (4.39E-04) and children (1.43E-04) inhaled airborne particles (0.1–0.5 μm) containing heavy metals, the carcinogenesis risk was higher than the risk value (10^−4^), indicating a strong carcinogenic effect.

Overall, the health risk assessment of different particle sizes was analyzed, and we found that the hazard index (HI) and the total carcinogenic risk (TCR) were far beyond the safety threshold when the size of the particle size was 0.1–0.5 μm. The degree of heavy metals contained in other particle sizes (d < 0.1 μm, 0.5–1 μm, and 1–2.5 μm) was generally beyond the safety threshold. Therefore, the air quality in Dongguan was considered to be in a risk state. Children were at more non-carcinogenic and carcinogenic risk than adults during the winter season of 2016–2017.

## Summary

In this study, we analyzed urban atmospheric environmental particles with different diameters in the Dongguan area. We collected atmospheric fine particles in winter and analyzed the partitioning of the particle-bound metals and the sources of metals in the particulate matter and their biotoxic effects. We then assessed their non-carcinogenic and carcinogenic health risks. When we studied the concentration of the total metals in the atmospheric particulates, we found that the F1 and F4 components in the atmospheric particulates were indicators. When these two components increase, the concentrations of metals bound to atmospheric fine particles are more abundant, indicating that human pollution is the main cause. Elements such as As, Ba, Cd, Mg, and Pb show relatively high proportions in the F1 fraction and strong potentials for the bio-accessibilities of many metal fractions in the atmosphere, and appear to be enhanced by high temperatures and humidity. Mn and Cr showed the highest HQ values and had the highest carcinogenic-associated risk for both children and adults. The risk index (HI) and the carcinogenic risk (TCR) were far beyond the safety threshold when the particle size was 0.1–0.5 μm. Our results indicate that the residents in Dongguan city are experiencing obvious non-carcinogenic and carcinogenic risks.
